# Neutrophils Induce a Novel Chemokine Receptors Repertoire During Influenza Pneumonia

**DOI:** 10.3389/fcimb.2019.00108

**Published:** 2019-04-16

**Authors:** Jennifer M. Rudd, Sivasami Pulavendran, Harshini K. Ashar, Jerry W. Ritchey, Timothy A. Snider, Jerry R. Malayer, Montelongo Marie, Vincent T. K. Chow, Teluguakula Narasaraju

**Affiliations:** ^1^Center for Veterinary Health Sciences, Oklahoma State University, Stillwater, OK, United States; ^2^Department of Microbiology and Immunology, Yong Loo Lin School of Medicine, National University of Singapore, National University Health System, Singapore, Singapore

**Keywords:** influenza, neutrophil, acute lung injury, chemokine receptor, mouse model

## Abstract

Exaggerated host innate immune responses have been implicated in severe influenza pneumonia. We have previously demonstrated that excessive neutrophils recruited during influenza infection drive pulmonary pathology through induction of neutrophil extracellular traps (NETs) and release of extracellular histones. Chemokine receptors (CRs) are essential in the recruitment and activation of leukocytes. Although neutrophils have been implicated in influenza pathogenesis, little is known about their phenotypic changes, including expression of CRs occurring in the infected -lung microenvironment. Here, we examined CC and CXC CRs detection in circulating as well as lung-recruited neutrophils during influenza infection in mice using flow cytometry analyses. Our studies revealed that lung-recruited neutrophils displayed induction of CRs, including CCR1, CCR2, CCR3, CCR5, CXCR1, CXCR3, and CXCR4, all of which were marginally induced in circulating neutrophils. CXCR2 was the most predominant CR observed in both circulating and lung-infiltrated neutrophils after infection. The stimulation of these induced CRs modulated neutrophil phagocytic activity, ligand-specific neutrophil migration, bacterial killing, and NETs induction *ex vivo*. These findings indicate that neutrophils induce a novel CR repertoire in the infectious lung microenvironment, which alters their functionality during influenza pneumonia.

## Introduction

Frequent outbreaks of influenza virus infections are causing significant morbidity and mortality in humans, birds, and other animal species (Xu et al., 2006; Traylor et al., 2013; Short et al., 2014; Wang et al., 2016). Neutrophils and macrophages constitute the majority of infiltrated cells in the lungs during influenza, and play essential roles in the clearance of the virus, before the onset of virus-specific immunity (Perrone et al., 2008; Tavares et al., 2017). However, uncontrolled recruitment and activation of these innate immune cells contribute to acute lung injury (ALI), significantly impacting the disease outcome (Crowe et al., 2009; Mauad et al., 2010; Liu et al., 2015). Our earlier studies have demonstrated that in severe influenza, the massive influx of neutrophils into the infected lungs causes collateral damage to the lungs *via* generation of NETs and the release of extracellular histones (Narasaraju et al., 2011; Anandi et al., 2013; Ashar et al., 2018).

The recruitment, extravasation, and activation of neutrophils are largely driven by chemokine ligands *via* binding to their cell-surface receptors called chemokine receptors (CRs) (Moser et al., 2004). CRs belong to a family of seven-transmembrane domain G protein-coupled receptors, divided into four structural groups (C, CC, CXC, and CX_3_C) based on the spacing of two conserved cysteine residues. Inflammatory chemokines produced in response to influenza by lung epithelial cells and/or macrophages regulate leukocyte recruitment and activation in infected lungs (Rossi, 2000; Moser and Loetscher, 2001). Neutrophils are generally thought to be limited in expression of CRs, typically consisting predominantly of the CXC group CRs (such as CXCR1, CXCR2); expression of CC chemokine receptors are absent under normal conditions (Sallusto et al., 2000). However, in inflammatory disease conditions such as rheumatoid arthritis, sepsis, and cystic fibrosis, neutrophils have been shown to expand their CR expression repertoire, especially after translocating into various tissues (Speyer et al., 2004; Hartl et al., 2008; Chou et al., 2010; Lebre et al., 2011). Induction of these CC CRs significantly alters neutrophil function, including phagocytosis, respiratory burst, and chemotaxis (Hartl et al., 2008; Chou et al., 2010). Neutrophils isolated from influenza-infected patients display impaired expression of phagocytic receptors such as CD64, CD32, and CD16, indicating that influenza infection modulates neutrophil functionality, which may also contribute to increased susceptibility to bacterial superinfections (Salentin, 2003). Influenza infection also modulates expression and chemotactic responsiveness of CCR1 and CCR2 in monocytes (Pauksens et al., 2008). Following excessive neutrophil influx, their toxic products such as NETs and granule enzymes are associated with pulmonary pathology in influenza pneumonia, although little is known about the phenotypic and functional characteristics of these neutrophils (Narasaraju et al., 2011; Anandi et al., 2013;Rojas-Quintero et al., 2018).

Here, we investigated whether hyper-inflammatory cytokine responses seen during influenza pneumonia alters the phenotypic signature of CR induction in lung-recruited neutrophils. Using Flow cytometry analysis, we have evaluated cell surface receptor expression of CRs (including CC and CXC types) in circulating as well as lung-recruited neutrophils during the course of infection. We evaluated the effects of induced CRs on neutrophil functionality, including phagocytosis, neutrophil migration, bacterial killing, and NETosis. Our results demonstrated induction of various CC and CXC-type CRs in neutrophils after their recruitment into the infected lungs, but not while in circulation. Further, activation of induced CRs with their specific chemokine ligands modulates neutrophil functional activities including phagocytosis, neutrophil migration, and NETosis. These studies suggest that induction of various CRs in lung-recruited neutrophils shape their fate and functional responsiveness in influenza infected-lungs.

## Materials and Methods

### Virus, Animals, and Ethics Approval

Influenza A/Puerto Rico/8/34, H1N1 (PR/8) virus was obtained from the American Type Culture Collection (ATCC, VA). Viral titers were determined by tissue culture infectivity dose (TCID_50_) assay via infection of Madin-Darby canine kidney (MDCK) cells (Ng et al., 2012). Female BALB/c mice (6–8 weeks old) were used in this study. The animals were housed in microisolator cages in a BSL-2 animal facility. All animal experiments were approved by the Institutional Animal Care and Use Committee (IACUC) of Oklahoma State University (protocol number VM-11-43) and were performed in strict accordance with their recommendations.

### Animal Infections

For influenza infections, mice were anesthetized with a mixture of xylazine (0.1 mg/kg) and ketamine (7.5 mg/kg). Mice were infected intranasally (IN) with a sub-lethal dose (100 TCID_50_) of PR/8 (H1N1) influenza virus in a 50 μL volume of sterile phosphate-buffered saline. Control mice received equal volumes of PBS.

### Collection of Blood, Bronchoalveolar Lavage (BAL) Fluid, and Tissues

For BAL fluid collection, the lungs were lavaged twice using intratracheal administration of 0.5 mL of sterile PBS (Ashar et al., 2018). The recovery of BAL fluid was over 85% for all animals. The BAL fluid samples were centrifuged at 200 x g for 10 min, and BAL cells were resuspended in sterile PBS containing 2% fetal bovine serum for flow cytometry analysis. For differential cell counts, BAL cells were processed onto microscopic slides using a CytoFuge 2 cytocentrifuge (StatSpin, Westwood, MA), subjected to modified Giemsa staining, and cells (more than 200 per animal) were counted at a magnification of 1000x. Whole blood was obtained via terminal procedure of intra-cardiac collection. BAL and blood were collected from control and infected mice at 3, 4, and 5 days post-infection (dpi) for flow cytometry analysis (we found that neutrophils influx peaked from 3 dpi). To exclude that inflammatory responses were due to secondary bacterial infection, 20 μL of each BALF sample was plated onto blood agar and incubated at 37°C for 3 days. In another set of experiments, control and infected animal lungs were fixed with 4% formalin, and subjected to histopathology analysis after hematoxylin and eosin (H&E) staining to evaluate inflammatory and acute lung injury. Histopathologic severity was scored in a blinded fashion on a scale of 1–4 (four being the most severe) based on the following criteria by a board-certified anatomic veterinary pathologist: cellular inflammation, necrotizing bronchiolitis, interstitial pneumonia, alveolitis, hemorrhage, and edema. Total histopathologic scores were evaluated as a sum of all individual scores (Narasaraju et al., 2010).

### Flow Cytometry Analyses

The following mouse antibodies were purchased from R&D Systems and used for flow cytometry for detection of chemokine receptors in neutrophils: CCR1 FITC-conjugated antibody (Clone 643854), CCR2 PE-conjugated antibody (Clone 475301), CCR3 PE-conjugated antibody (Clone 83101), CCR5 FITC-conjugated antibody (Clone CTC5), CXCR1/IL-8 RA PE-conjugated antibody (Clone 1122A), CXCR2/IL-8 RB PE-conjugated antibody Clone 242216), CXCR3 PE-conjugated antibody (Clone 220803), CXCR4 fluorescein-conjugated antibody (Clone 247506), and Ly6G-1A8 PerCP-conjugated antibody (Clone 1A8) were purchased from BioLegend, CA (Hartl et al., 2008).

Phenotypic characterization of neutrophils during the course of infection was performed using whole blood from control mice; blood and BAL cells from influenza-infected mice at 3, 4, and 5 dpi. Control BAL samples were not used as they contained very low numbers of neutrophils to perform flow cytometry. Blood and BAL samples were incubated with RBC lysis buffer (Miltenyi Biotec Inc, CA), followed by incubation with chemokine receptor specific antibodies for 30 min at room temperature. Unstained cells and single-fluorochrome staining controls were used to exclude background and cross-reactivity among different fluorochromes. All samples were then centrifuged and washed thrice with PBS (containing 2% fetal bovine serum) before performing flow cytometry. The latter was performed using BD FACSCalibur flow cytometer, and the data were analyzed using CellPro software. Neutrophils were gated as Ly6G-1A8^+^SSC^med-hi^. CD11b analysis was performed by comparing mean fluorescence intensity (MFI) between samples. The Ly6G-1A8 staining on neutrophils was validated by sorting Ly6G-1A8 positive cells on FACSAria flow cytometer, which displayed 99% purity. All flow cytometry experiments were repeated three times, and in each experiment, cells were prepared from a pool of three mice to obtain sufficient numbers of cells.

### Neutrophil Isolation

For neutrophil functional analysis, neutrophils were isolated from control blood, infected blood, and BAL samples using a MACS neutrophil isolation kit (Miltenyi Biotec Inc, CA) with Ly6G-1A8 antibody through positive selection (Ashar et al., 2018). Isolated neutrophils were enumerated and used for functional studies. Blood samples were initially incubated with 1x RBC lysis buffer to remove RBCs, prior to isolation of neutrophils.

### Phagocytosis Assay

For phagocytosis assays, BAL neutrophils were isolated, and 10^5^ cells were stimulated with or without the appropriate CCR1, CCR3, CCR5, CXCR2, CXCR3, and CXCR4 blockers and ligands. One microgram of BX 471 (CCR1 Antagonist; Cayman Chemicals, MI), SB328437 (CCR3 Antagonist; Sigma, MN), anti-CCR5 (Novus, CO), anti-CXCR2 (Cell Applications, CA), CXCR3 (Bio X Cell, NH) blocking antibodies, and AMD3100 (CXCR4 Antagonist (R&D, MN) were added and incubated for 30 min at 37°C. The cells were then stimulated with 10 ng of the appropriate ligands CCL3 (CCR1), CCL11 (CCR3), CCL4 (CCR5), IL-8 (CXCR2), CXCL11 (CXCR3), and CXCL12 (CXCR4). pHrodoTM Red *E. coli* BioParticles (Thermo Fisher, MA) were added to each sample (1 mg/mL), and cells were incubated at 37°C for 90 min. Cells were then stained with Ly6G-1A8 antibody for 30 min at room temperature, washed twice to remove excess bacteria, followed by flow cytometry (Hartl et al., 2008). Results were analyzed by determining MFI. Unstained, single-stained neutrophils and bacteria alone served as controls.

### Neutrophil Chemotaxis Assay

Neutrophil chemotaxis assay was performed as described by Szczur et al. (2006). In brief, BAL neutrophils isolated at 4 dpi were purified, resuspended in DMEM containing 1% fetal bovine serum and added to (1 × 10^5^/well) the upper compartment of a Transwell filter system (8.0 μm pore size, 12 mm diameter) in a 24-well culture plate. The chemokine specific ligands including CCL3, CCL4 (R&D), IL-8, and CXCL11 (R&D) (at a concentration of 100 ng/ml) were added to the lower chamber. The plate was incubated for 90 min at 37°C. The culture medium from the lower chamber was centrifuged, and the migrated cells were counted with hemocytometer (Szczur et al., 2006).

### *In vitro* NETs Release

To test the effect of various chemokine receptors on NETosis, neutrophils isolated from influenza-infected lungs at 4 dpi were resuspended in DMEM containing 1% fetal bovine serum and stimulated (2 × 10^4^) with chemokine specific ligands including CCL4, IL-8, and CXCL11 as described above. Phorbol 12-myristate 13-acetate (PMA) at 20 nM concentration was used as a positive control for induction of NETs (Ashar et al., 2018). Released NETs were labeled with SYTOX green staining and visualized under fluorescence microscopy at 400x magnification. Quantification of NETs released was performed as described earlier (Ashar et al., 2018). We evaluated at least 5–10 fields on each slide to quantify the total numbers of positive cells exhibiting NETs release.

### Bactericidal Activity

Neutrophils (105) isolated from influenza-infected mice at 4 dpi were incubated with *Streptococcus pneumoniae* at 1:10 ratio for 90 min in the presence or absence of CCL3, CCL4, IL-8, CXCL11 (100 ng/mL). Bacterial killing was measured as a percentage of control bacteria (bacteria incubated without neutrophils) as described previously (Narasaraju et al., 2011). Sample aliquots were plated on chocolate agar to determine the numbers of colony-forming units (CFU).

### Statistical Analysis

The data are expressed as the means ± SEM. Statistical analyses were performed using Student's unpaired *t*-test, paired *t*-test or analysis of variance (ANOVA) using GraphPad Prism 7 software. A value of *p* < 0.05 was considered as statistically significant.

## Results

### Lung-Infiltrated Neutrophils Induce Novel Chemokine Receptors During Influenza-Infection

**Figure 1:**
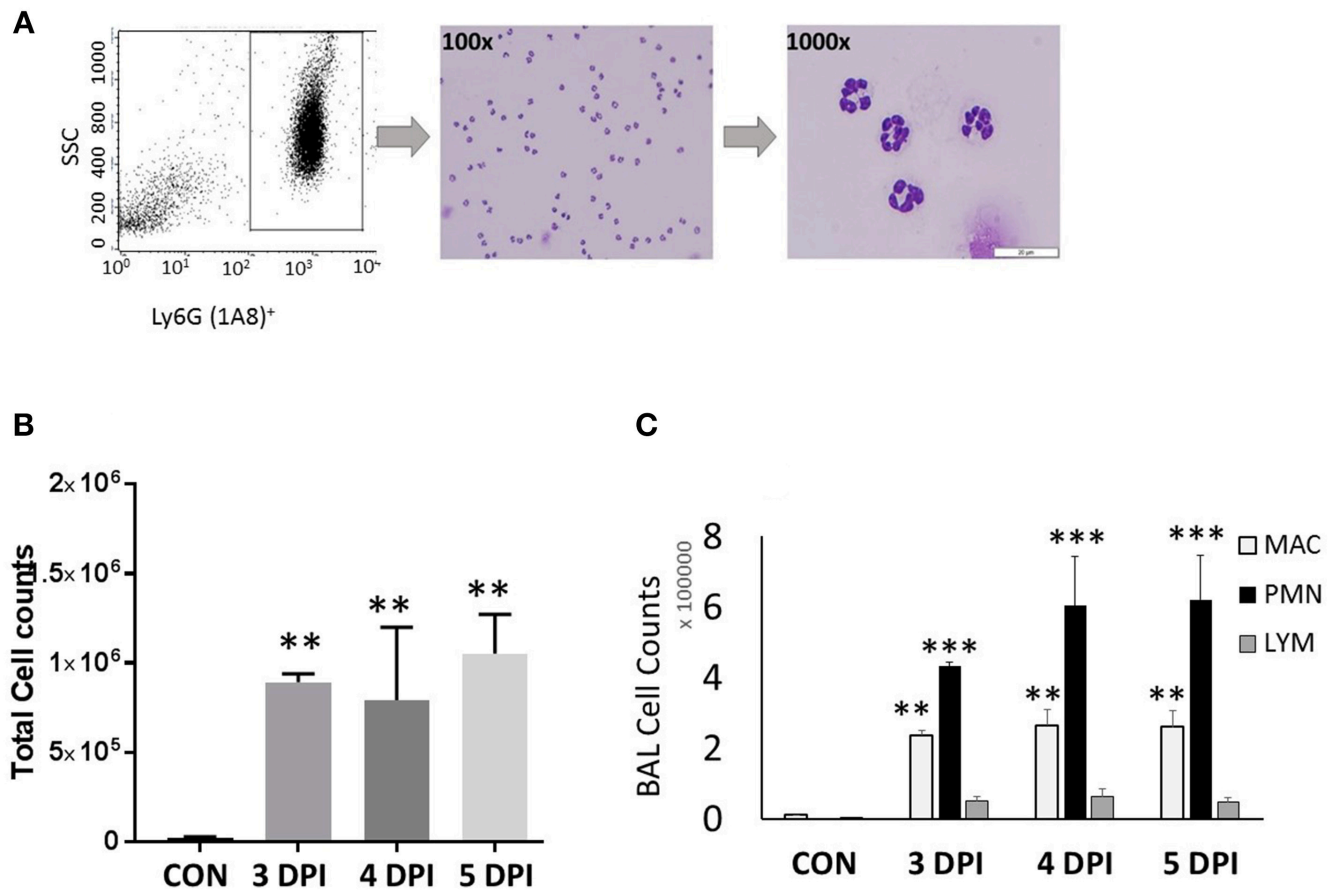
Neutrophil gating and differential counts during influenza infection. BALB/C mice were infected with a sub-lethal dose (100 TCID_50_), intranasally with influenza A/Puerto Rico/8/34 H1N1 virus. Mock-infected mice received equal volumes of PBS. **(A)** Identification of neutrophils and gating. Neutrophils were identified based on their light scatter characteristics (FSC/SSC). The granulocyte region was further differentiated by means of the neutrophil specific marker, Ly6G-1A8. FSC^med^SSC^med-*hi*^Ly6G-1A8^+^ cells were further sorted using a FACSAria flow cytometer, which showed that over 99% were neutrophils. Morphologically, neutrophils were identified by modified Giemsa staining. The representing images are showing neutrophils at 100x and 1000x. **(B)** Influenza-infected mice have significantly elevated BAL leukocytes between 3 and 5 dpi. **(C)** Differential cell counts were performed in lung-recruited cells, and revealed neutrophils as the major cell population. Data were expressed as means ± SEM. *n* = 3–5 mice per group; ^**^p < 0.01; ^***^p < 0.001 vs. control.

We have evaluated various CC and CXC chemokine receptors in circulating neutrophils as well as lung-recruited neutrophils in influenza-infected mice. Neutrophils were gated based on their FSC/SSC characteristics, followed by detection of Ly6G-1A8 on 10,000 events. Neutrophils, SSC^med-hi^/Ly6G-1A8^+^ were separated by a cell sorter, resulting in 99% purity based on modified Giemsa staining (Figure 1A). We then evaluated induction of a broad range of CRs, including CC (CCR1-3, CCR5) and CXC (CXCR1-4), by flow cytometry analysis using control blood, infected blood, and BAL. Control BAL cells were not included in this study as they contained too few neutrophils to perform flow cytometry. We characterized chemokine receptor profiles in neutrophils between 3 and 5 dpi, which displayed persistent increase of these cells into the infected-lungs (Figures 1B,C), with vascular injury evident by protein leakage and total histopathologic changes in the lungs (**Figures 6A–C**).

**Figure 2:**
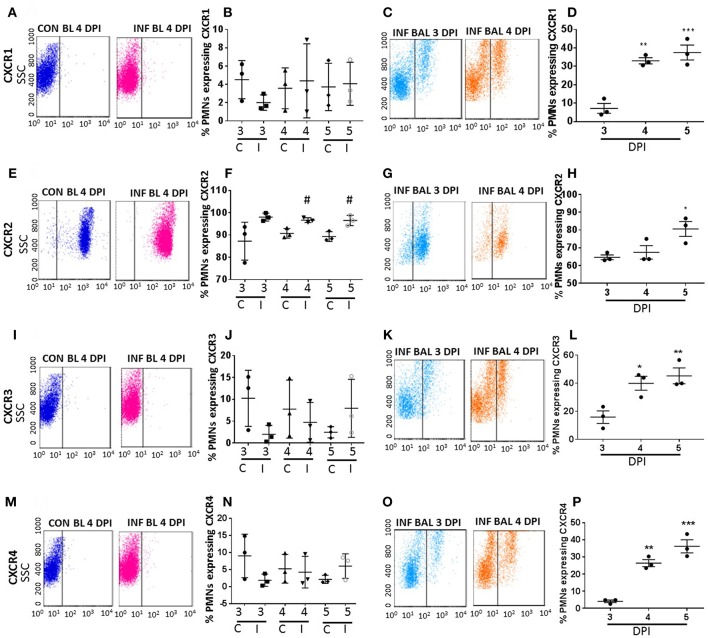
Surface CXC-chemokine receptor detection profiles in blood and lung-recruited neutrophils during influenza infection. Blood from control and influenza-infected mice at 3–5 dpi were assessed by flow cytometry. Neutrophils were gated as Ly6G-1A8^+^ cells for subsequent analysis. Induction of receptors was represented as percentage of blood neutrophils showing positive staining for chemokine receptors CXCR1 **(A,B)**, CXCR2 **(E,F)**, CXCR3 **(I,J)**, and CXCR4 (**M,N)**. Lung-recruited neutrophils were analyzed at 3,4, and 5 dpi for induction of CXCR1 **(C,D)**, CXCR2 **(G,H)**, CXCR3 **(K,L)**, and CXCR4 **(O,P)**. Dot plots represent detection of CXCR1, CXCR2, CXCR3, and CXCR4 in control blood, infected blood (4 dpi) and infected BAL (3 and 4 dpi), and the subsequent graph represents the overall trend in percentage induction of receptor on neutrophils in all samples from 3 through 5 dpi. Data are expressed as mean ± SEM. *n* = 3 replicates and each replicate prepared from a pool of three mice for all receptor expression analysis. ^#^*p* < 0.05; vs. Con blood. ^*^p < 0.05; ^**^p < 0.01; ^***^p < 0.001 vs. 3 dpi BAL. C-Con, I-Infected.

**Figure 3:**
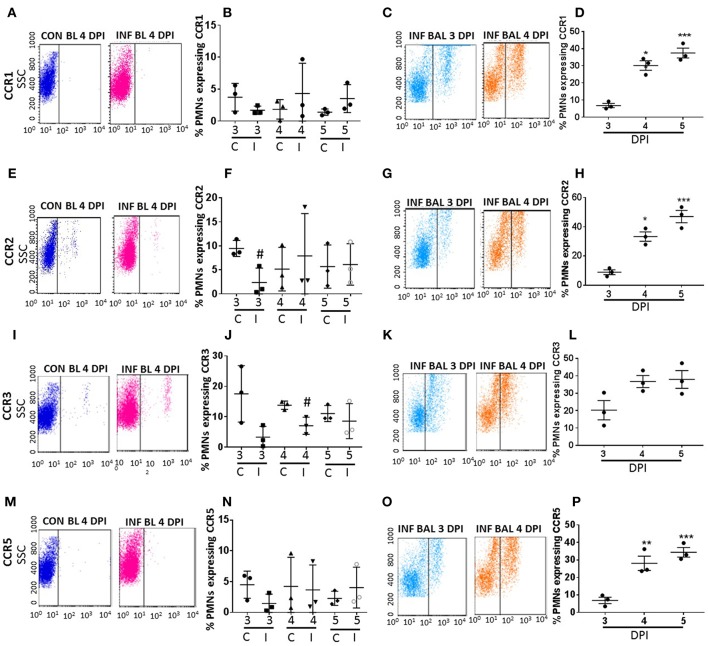
Surface CC-chemokine receptor detection in blood and lung-recruited neutrophils during influenza infection. Blood from control and influenza-infected mice at 3–5 dpi were assessed by flow cytometry. Neutrophils were gated as Ly6G-1A8^+^ cells for subsequent analysis. Detection of receptors was represented as percentage of neutrophils showing positive staining for chemokine receptors CCR1, CCR2, CCR3, and CCR5. Detection of receptors was represented as percentage of blood neutrophils showing positive staining for chemokine receptors CCR1 **(A,B)**, CCR2 **(E,F)**, CCR3 **(I,J)**, and CCR5 **(M,N)**. Lung recruited neutrophils were analyzed at 3,4, and 5 dpi for detection of CCR1 **(C,D)**, CCR2 **(G,H)**, CCR3 **(K,L)**, and CCR5 **(O,P)** Dot blots represent expression of CCR1, CCR2, CXR3, and CCR5 in control blood, infected blood (4 dpi) and infected BAL (3 and 4 dpi). Data are expressed as mean ± SEM. *n* = 3 replicates and each replicate prepared from a pool of three mice for all receptor expression analysis. ^#^*p* < 0.05 vs. con blood. ^*^p < 0.05; ^**^p < 0.01; ^***^p < 0.001 vs. 3 dpi BAL.

**Figure 4:**
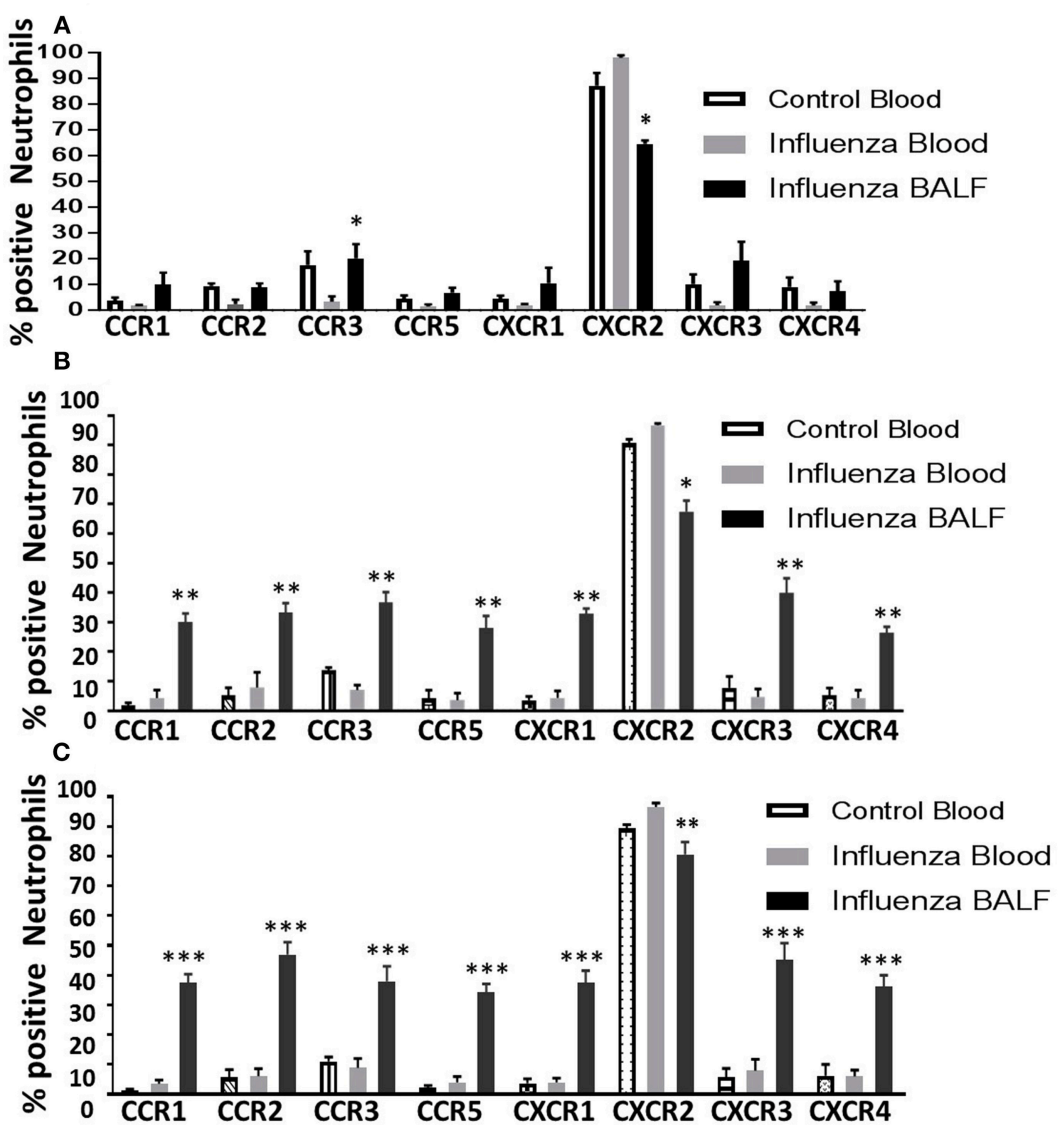
Overall induction of chemokine receptors in neutrophils during the course of infection. The surface detection of CC and CXC CRs were analyzed in a time-dependent fashion and compared with control blood neutrophils. Control blood, infected blood and infected BAL from mice at 3 dpi **(A)**; 4 dpi **(B)**, and 5 dpi **(C)**. Samples are represented as percentage expression on neutrophils compared between groups. Data are expressed as mean ± SEM. At each days post infection, was compared with infected (INF) blood samples to visualize the overall surface detection of CRs while in circulation and after recruiting into the infected-lungs. The control BAL samples were not included as they contained very low number of neutrophils to perform flow cytometry. Replicates (*n* = 3); each replicate was prepared from a pool of three mice for all receptor expression analysis. ^*^p < 0.05; ^**^p < 0.01; ^***^p < 0.001; vs. Infected blood at each day post infection.

To evaluate induction of various CRs, neutrophils from blood and BAL samples were labeled with CC (CCR1-CCR3, CCR5) and CXC (CXCR1-4) specific antibodies. Neutrophils were identified by Ly6G-1A8^+^ staining. Our studies revealed that majority of the CRs do not show significant increase while in circulation in infected-mice. However, the CRs induced in infected-lung microenvironment. Lung-recruited neutrophils induced CCR1, CCR2, CCR3, CCR5, CXCR1, CXCR3, CXCR4, which were absent or marginally induced in peripheral blood neutrophils from influenza-infected mice. Neutrophils from infected-blood showed minimal to absent of CC CRs similar to healthy mice samples. The expression pattern of majority of CC and CXC CRs was consistently elevated from 3 dpi through 5 dpi in lung-recruited neutrophils, but not while in circulation (Figures 2–**4**).

Among all CRs, CXCR2 was the most abundant CR detected in control blood, infected blood, and BAL neutrophils (Figures 2E–H). Circulating neutrophils from healthy control mice were 85% positive for CXCR2, which increased to 97–100% in infected blood samples (Figure 2F). Upon pulmonary infiltration in response to infection, these neutrophils exhibited decline in CXCR2-positive staining, but remained highly induced at over 60% positivity compared to other induced CRs in infected-lungs (**Figures 4A–C**). There was no difference in surface expression levels of CXCR1 in circulating neutrophils, but increased from 3 to 5 dpi with about 30–40% elevation in lung-recruited neutrophils at 4 and 5 dpi (Figures 2A–D). The detection of other CXC CRs (including CXCR3 and CXCR4) also displayed a similar trend in their surface expression during the course of infection between 3 and 5 dpi, while there was no difference in circulating neutrophils between control and infected groups (Figures 2I–P, **4A–C**).

The surface expression of CC CRs was minimal to absent in circulating neutrophils in both control and influenza-infected mice. However, CC CRs including CCR1, CCR2, CCR3, and CCR5 were significantly increased in lung-recruited neutrophils (Figures 3A–P). The induction of these chemokine were altered between control and infected blood groups (Figures 3B,F,J,N). These CC CRs were present in about 10–15% of BAL cells at 3 dpi, and increased to 20–30% at 4 dpi and to 30–40% at 5 dpi (Figures 3D,H,L,P, 4B,C), suggesting that the inflammatory cytokine environment significantly modulates neutrophil chemokine receptor induction during influenza pneumonia.

### Effect of Induced Chemokine Receptors on Phagocytic Activity in Lung-Recruited Neutrophils Following Influenza Infection

**Figure 5:**
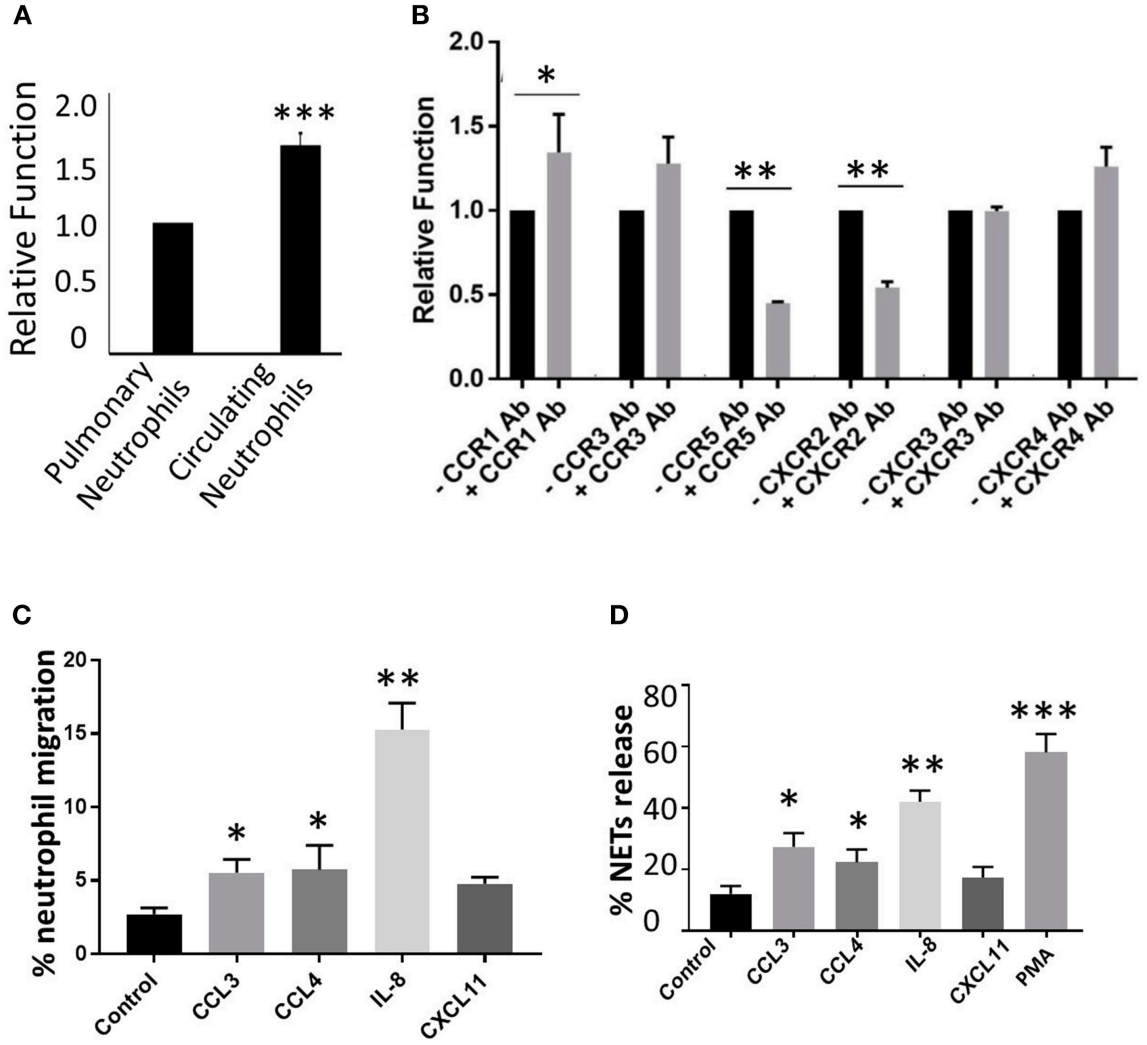
Effect of CR induction on neutrophil phagocytic activity. To test overall phagocytic activity between circulating and lung-recruited neutrophils, we isolated neutrophils from infected blood and BAL, and tested their phagocytic activity. **(A)** Neutrophils isolated from BAL exhibited diminished phagocytic capacity compared with those in circulation. **(B)** Phagocytic activity using CCR1, CCR3, CCR5, CXCR2, CXCR3, and CXCR4 blockers and ligands. BX 471 (CCR1 antagonist), SB328437 (CCR3 antagonist), anti-CCR5, anti-CXCR2, CXCR3 blocking antibodies, and AMD3100 (CXCR4 antagonist) were added and incubated for 30 min at 37°C. The cells were then stimulated with the appropriate ligands CCL3 (CCR1), CCL11 (CCR3), CCL4 (CCR5), IL-8 (CXCR2), CXCL11 (CXCR3), and CXCL12 (CXCR4). Blocking CCR5 and CXCR2 reduced phagocytic capacity of pulmonary infiltrating neutrophils in influenza viral infection, while no change was observed with CXCR3 blockade. In contrast, CCR1 inhibition resulted in enhanced phagocytic activity. **(C)** Neutrophil chemotaxis assay was performed by stimulating neutrophils isolated from infected BAL using CCL3 (CCR1), CCL4 (CCR5), IL-8 (CXCR2), CXCL11 (CXCR3). Data are represented as percentage migration. **(D)** NETosis was evaluated by stimulating neutrophils isolated from infected BAL using CCL3 (CCR1), CCL4 (CCR5), IL-8 (CXCR2), CXCL11 (CXCR3). Data are represented as percentage of NETs formation. Mean ± SEM. *n* = 3 independent experiments, and cells were collected by pooling two mice in each experiment. ^*^p < 0.05; ^**^p < 0.01; ^***^p < 0.001.

Earlier studies have shown that impaired phagocytic activity during influenza (Ishikawa et al., 2016), and lung-recruited neutrophils fail to kill bacteria *in vivo* (Hashimoto et al., 2007). We found that overall phagocytic activity declined in lung-recruited neutrophils compared to circulating neutrophils (Figure 5A). Next, to test if the induced CRs in lung-recruited neutrophils contribute to phagocytic function in infected-lungs, we stimulated upregulated CRs in lung-recruited neutrophils with specific chemokine ligands in the presence or absence of CCR1, CCR3, CCR5, CXCR2, CXCR3, and CXCR4 specific blocking antibodies. Our results demonstrated that antibody blocking of CCR5 and CXCR2 significantly inhibited phagocytic activity. Interestingly, blockade with CCR1 antibody revealed enhanced phagocytic activity (Figure 5B). No significant differences in phagocytic activity was observed when CCR3, CXCR3, and CXCR4 were blocked. Based on these findings, we used ligand-specific stimulation for CCR1, CCR5, CXCR2, and CXCR3 to test their effects on neutrophil functional responsiveness including chemotaxis, bacterial killing, and NETosis.

### Effect of Induced CRs on Neutrophil Chemotaxis

Neutrophils isolated from influenza-infected mouse lungs were seeded in the upper chamber of the 8.0-μm membrane insert in a Transwell system. CCR1, CCR5, CXCR2, and CXCR3 specific ligands including CCL3, CCL4, IL-8, and CXCL11 (100 ng/mL) were added into the lower chamber and incubated for 90 min. Incubation with IL-8 culminated in a 4-fold increase in neutrophil migration. The addition of ligands including CCL4, and CCL3, but not CXCL11 demonstrated ~2-fold enhancement in neutrophil migration (Figure 5C).

### Induced CRs Modulate Release of NETs in Lung-Recruited Neutrophils *in vitro*

Neutrophils isolated from influenza-infected mouse lungs were stimulated with CCR1, CCR5, CXCR2, and CXCR3 specific ligands CCL3, CCL4, IL-8, and CXCL11 (100 ng/mL), respectively, and incubated for 4 h. NETs were stained with SYTOX green (Ashar et al., 2018). Stimulation of neutrophils with IL-8 (CXCR2 ligand) generated pronounced release of NETs. Significantly elevated NETosis was also observed when neutrophils were stimulated with CCL3 and CCL4. However, CXCL11 did not lead to prominent NETosis (Figure 5D).

### Stimulation of Induced CRs Does Not Enhance Bacterial Killing

Neutrophils isolated from influenza-infected mouse lungs were stimulated with CCR1, CCR5, CXCR2, and CXCR3 specific ligands including CCL3, CCL4, IL-8, and CXCL11 (100 ng/mL) for 20 min, followed by incubation with a 1:10 ratio of *Streptococcus pneumoniae* (serotype 3) for 90 min. No difference in bacterial numbers was observed in cells stimulated with any of these chemokine ligands compared to the bacteria-alone group, thus indicating that activation of these CRs do not interfere with bactericidal activity of neutrophils (data not shown).

### Influenza Infection of Lungs Leads to Excessive Neutrophil Influx and Widespread Pulmonary Damage

**Figure 6:**
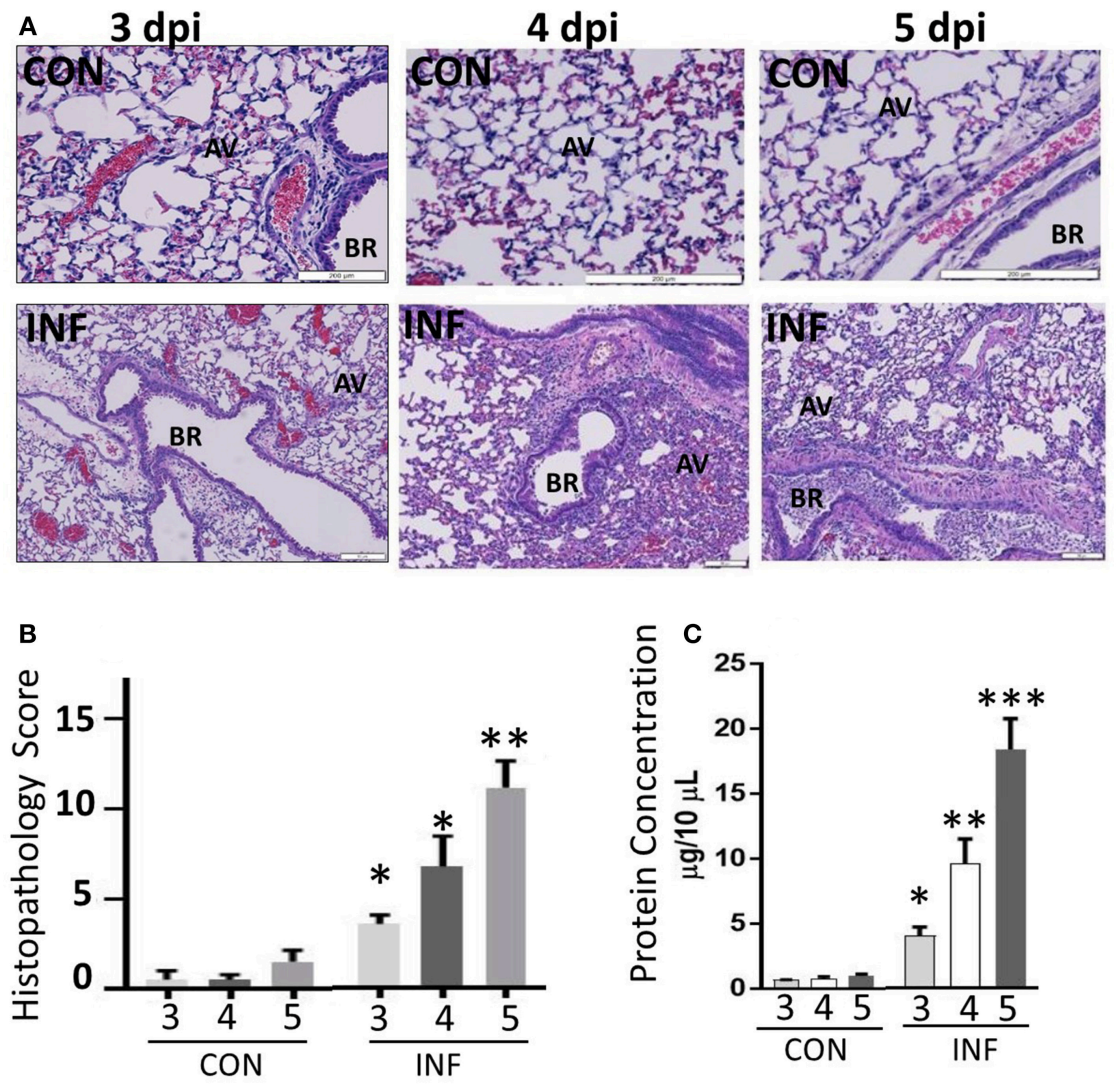
Influenza infection increases inflammation and severe pulmonary pathology. **(A)** Paraffin-embedded lung tissues from 3 to 5 days following challenge with infection or mock infection were stained with hematoxylin and eosin. Infected lungs displayed the highest severity score with notable pulmonary edema, bronchiolitis, alveolitis, hemorrhage, and interstitial disease. **(B)** Total histopathologic scores of infected samples were compared with controls. Data were expressed as means ± SEM. *n* = 4 mice per group. **(C)** Vascular leakage was determined by measuring total proteins present in the BAL fluid samples collected from control and influenza-infected mice at 3, 4, and 5 dpi. AV, alveoli; BR, bronchioles. ^*^p < 0.05; ^**^p < 0.01; ^***^p < 0.001.

We performed histopathologic analysis to test for a correlation between neutrophilic inflammation and pathologic lesions between 3 and 5 dpi. Neutrophil-influx was significant and comparable between 3 and 5 dpi, while the changes in neutrophil phenotypic support increase in pathologic lesions with augmented alveolar injury, vascular damage and bronchiolitis (Figures 6A,B). Our studies indicate a significant increase in lung pathology. BAL fluid cell counts performed on days 3–5 dpi also displayed an increase in total cell numbers. In support of this, we found significant vascular leakage from 3 to 5 dpi (Figure 6C). Further, we did not find any bacterial growth from the BAL samples from influenza-infected mice (data not shown), indicating that neutrophil inflammation or induction of CR are not due to bacterial superinfection.

## Discussion

Newly emerging and re-emerging influenza virus infections remain a continuous threat worldwide. Influenza infections trigger hyper-inflammatory cytokine responses together with rapid, massive cellular influx, predominantly by neutrophils, and macrophages (de Jong et al., 2006; Perrone et al., 2008; Taubenberger and Morens, 2008). We have previously shown that overly exuberant neutrophils produce NETs and extracellular histones which disrupt the alveolar-capillary barrier, resulting in alveolar injury and vascular leakage (Narasaraju et al., 2011; Ashar et al., 2018). Neutrophils are short-lived and terminally differentiated innate immune cells with primary roles in phagocytic clearance of influenza-infected cells. Although exaggerated neutrophil recruitment and their activation are linked to acute lung pathology during influenza, little is known about their phenotypic or functional characteristics (Kobasa et al., 2004; Tumpey et al., 2005; Taubenberger and Morens, 2008; Yokoyama et al., 2010). Here, we provide evidence that lung-recruited neutrophils expand their CR repertoire during influenza infection of lungs. Lung-sequestered neutrophils displayed up-regulation of several CRs (such as CCR1, CCR2, CCR3, CCR5, CXCR1, CXCR3, and CXCR4) that are minimally expressed or absent while in circulation. The surface induction of these CRs increased in a time-dependent manner in pulmonary-recruited neutrophils. Furthermore, induced CRs in lung-recruited neutrophils potentially modulate neutrophil functions, including chemotaxis, phagocytosis, and NETosis. These results indicate that the infected-lung microenvironment significantly affects neutrophil phenotypic signature and their functional responsiveness, and these changes could considerably impact the disease pathogenesis in influenza pneumonia.

Neutrophils conventionally express CXC chemokine receptors, while CC chemokine receptors are generally absent and unresponsive to CC chemokine ligand stimulations. However, studies have shown that neutrophils isolated from lungs or synovial cavities from patients with chronic obstructive pulmonary disease (COPD), rheumatoid arthritis or sepsis (Speyer et al., 2004; Hartl et al., 2008; Chou et al., 2010; Lebre et al., 2011) display induced expression of CC CRs, and that pro-inflammatory cytokines including IFN-γ, TNF-α, and GM-CSF modulate expression of these CRs. Further, the induced CRs alter neutrophil functions, including respiratory burst, degranulation, and chemotaxis thus contributing to inflammation and injury (Hartl et al., 2008). Hyper cytokine responses, also termed as the “cytokine storm” are associated with pulmonary pathology in fatal influenza pneumonia (de Jong et al., 2006; Teijaro et al., 2014; Guo and Thomas, 2017). Influenza primarily infects lung epithelial cells and macrophages, which trigger pro-inflammatory cytokines induction and activation of various toll-like receptors (TLRs) and retinoic acid-inducible receptors (RIG-1)-mediated signaling, leading to persistent elevation in cytokines/chemokines in infected-lungs culminating in immunopathology (Wang et al., 2008; Shirey et al., 2013; Iwasaki and Pillai, 2014; Pulendran and Maddur, 2015; Kandasamy et al., 2016). We found early induction of chemokine ligands such as CCL4, CCL7, CCL2, CCLL, CXCL1, CXCL11, CXCL13 in infected mouse lungs (Ivan et al., 2012). Indeed early induction of pro-inflammatory cytokine response is detrimental in severe influenza pathogenesis (Perrone et al., 2008). Despite evidence demonstrating extensive cytokine induction in severe influenza pneumonia, little is known whether these secreted cytokines regulate induction of CRs in lung-infiltrated neutrophils. It is noteworthy that although neutrophils numbers peaked by 3 dpi, the induction of new CRs appeared up-regulated only from 4 dpi, indicating that induction of these CRs occurs under “cytokine/chemokine stress,” which could be critical in shaping the phenotype and functionality of neutrophils in the influenza-infected lung microenvironment.

Among CXC CRs, CXCR2 is the most abundantly expressed in circulating and lung-recruited neutrophils. The surface expression of CXCR2 is regulated by at least two mechanisms, including the receptor internalization/recycling or metalloprotease activity by a disintegrin and metalloproteinase domain-containing protein 1**7** (ADAM-17) (Mishra et al., 2014). Interestingly, percent neutrophils expressing CXCR2 was decreased in lung-recruited neutrophils at 3 dpi, compared to circulating neutrophils. However, CXCR2 surface expression increased significantly between 3 and 5 dpi. Similar to these findings, reduction in CXCR2-positive neutrophils is observed in patients with chronic inflammatory conditions (Hartl et al., 2008). The changes in surface expression of CXCR2 in blood and lung-recruited neutrophils may be attributed to receptor internalization and recycling upon increase in ligand-specific interaction in acute influenza infection (Mishra et al., 2014). Persistent increase in neutrophil influx and CXCR2 surface expression indicate that targeting CXCR2 could alleviate excessive neutrophils influx and lung pathology. Indeed, blocking CXCR2 has shown to reduce acute lung injury and inflammation in influenza-infected mice (Tavares et al., 2017) and mice lacking CXCR2 gene have shown decreased inflammation, without affecting viral clearance indicating pathogenic role of neutrophils in severe influenza (Wareing et al., 2007).

The functional significance of the induced CRs was investigated through chemokine-specific ligand activation and/or antibody blocking for neutrophil phagocytosis, chemotaxis, bacterial killing, and NETosis. Our studies revealed highly variable responses to different chemokines that are upregulated during infection. Blocking CCR5 and CXCR2 resulted in reduced phagocytic activity compared to the ligand-mediated stimulation, whereas CCR1 blockade augmented phagocytic activity. Blocking CCR3, CXCR1, CXCR3, and CXCR4 did not modify phagocytic activity. These findings are in partial agreement with previous findings of LPS-injury models, chronic inflammatory diseases in humans, which show that induced CC CRs (such as CCR1, CCR2, CCR3, CCR5) enhance phagocytic activity, and respiratory burst functions (Hartl et al., 2008; Wang et al., 2011). In contrast to the earlier reports, blocking CCR1 enhanced phagocytic activity. However, stimulation of CCR1 enhanced NETosis and chemotaxis, thus indicating that induced CRs exhibit differential functional responsiveness during influenza. Based on the phagocytic functions of different CRs, we tested the effects of induced CCR1, CCR5, CXCR2, and CXCR3 on neutrophil functions including chemotaxis, bacterial killing, and NETosis. Although CXCR2 is a critical CR that regulates neutrophil chemotaxis, and NETosis, induction of CCR5 and CCR1 also significantly impacted these neutrophil functions. Mice deficient of CCR5 gene are more susceptible to influenza infection and exhibit greater neutrophil influx compared to wild-type mice. However, deletion of CXCR3 together with CCR5 (CCR5^−/−^/CXCR3^−/−^) does not alter neutrophil influx. These findings are congruent with our findings that stimulation of CXCR3 does not significantly alter neutrophil migration or NETosis (Fadel et al., 2008).

Interestingly, none of the induced CRs show potential bactericidal effects, when we incubated neutrophils in the presence of *Streptococcus pneumoniae*, which is one of the commonest pathogens causing co-infections during influenza outbreaks (Kash et al., 2011; Moorthy et al., 2016). These results validate our earlier findings that neutrophils from influenza-infected mice lack bactericidal effects. It is noteworthy that close proximity of induced chemokine ligands with enhanced CRs have high probability to augment phagocytic function. However, the lack of *in vivo* bacterial killing suggests that lung-recruited neutrophils may engulf bacteria, but may be defective in bactericidal activity, which was evident from a report demonstrating increased neutrophils containing labeled bacteria (Ishikawa et al., 2016), but fail to kill the pathogen. The lack of bactericidal activity may also be attributed to impaired free radical generation. Influenza infection has also shown to impair NADPH oxidase activity (Sun and Metzger, 2014). A study has shown that seasonal and pandemic influenza viruses differentially regulate neutrophil respiratory burst and phagocytosis (Malachowa et al., 2018). The ability of influenza virus to impair phagocytic function may be due to the inhibition of azurophilic granules with the lysosomes during phagocytosis, thus preventing bacterial killing (Abramson et al., 1982). These findings support our earlier studies showing that massive neutrophil influx during influenza does not reduce bacterial loads. On the other hand, alveolar-capillary injury inflicted by NETs and extracellular histones may facilitate bacterial adhesion and growth, and thus exacerbate pulmonary pathology. Another study has shown that neutrophils also limit pulmonary pathology by suppressing T-cell mediated damage during influenza (Tak et al., 2018). The contribution of neutrophils to protection or injury may ultimately be dependent upon the neutrophil numbers and inflammatory cytokine responses. The virulence of the influenza virus strain may also influence the neutrophil functionality and pathogenesis, which warrants further investigations into the effects of different viral strains of varying pathogenicity on the cytokine storm and neutrophil phenotypic changes. These studies attempted to characterize and compare circulating as well as lung-recruited neutrophils. It would thus be interesting to evaluate neutrophils that are present within the lung parenchyma of infected lungs to determine if induction of CRs is also modified during transmigration of neutrophils from the circulation into the alveolar air space.

In conclusion, this study indicates an induction of CRs occurs upon neutrophil extravasation and activation into the pulmonary environment in a murine model of influenza pneumonia. These induced CRs could serve as potential therapeutic targets for alleviating neutrophil-induced lung pathology. Among all CRs, CXCR2 is most highly induced, and represents a promising target for therapy to reduce neutrophil recruitment to the area of inflammation. The functional properties of these individual chemokine receptors warrant further investigation to further understand how these induced CRs impact deleterious or beneficial effects of neutrophils as well as their roles in the context of influenza-induced acute lung injury.

## Ethics Statement

IACUC, Oklahoma State University. Protocol No: VM-17-32.

## Author Contributions

JMR, SP, HA, VC, and TN: conception and design of experiments; JMR, SP, HA, JWR, MM, and TN: acquisition and analysis of data; JRM, VC, and TN: critical review; TS: histopathology analysis.

### Conflict of Interest Statement

The authors declare that the research was conducted in the absence of any commercial or financial relationships that could be construed as a potential conflict of interest.
